# Peroral endoscopic myotomy provides effective and sustained relief for achalasia in Allgrove syndrome: a long-term comparative cohort study

**DOI:** 10.3389/fmed.2026.1819125

**Published:** 2026-04-17

**Authors:** Yuhao Liu, Yaowen Hu, Tao Guo, Zhifeng Wang, Xiaohong Sun, Aiming Yang, Yunlu Feng, Qingwei Jiang, Lin Lu, Xiaoqing Li

**Affiliations:** 1 Department of Gastroenterology, Peking Union Medical College Hospital, Chinese Academy of Medical Sciences and Peking Union Medical College, Beijing, China; 2 Department of Internal Medicine, Peking Union Medical College Hospital, Chinese Academy of Medical Sciences and Peking Union Medical College, Beijing, China; 3 Department of Geriatrics, Peking Union Medical College Hospital, Chinese Academy of Medical Sciences and Peking Union Medical College, Beijing, China; 4 Department of Endocrinology, Peking Union Medical College Hospital, Chinese Academy of Medical Sciences and Peking Union Medical College, Beijing, China

**Keywords:** *AAAS* gene, Allgrove syndrome, outcome, pediatric achalasia, peroral endoscopic myotomy

## Abstract

**Background:**

Allgrove syndrome is a rare autosomal recessive disorder characterized by the triad of alacrima, adrenal insufficiency, and achalasia. Although corticosteroid replacement and artificial tears effectively manage adrenal insufficiency and alacrima, the optimal treatment for achalasia in this syndrome remains challenging and poorly defined.

**Methods:**

All patients diagnosed with Allgrove syndrome were identified between 2009 and 2025 at Peking Union Medical College Hospital, which is a national rare disease center in China. Clinical characteristics and genetic mutations were collected and analyzed, and the long-term safety and efficacy of peroral endoscopic myotomy (POEM) in Allgrove syndrome were evaluated through follow-up telephone interviews. A comparison cohort of idiopathic achalasia (IAC) patients undergoing POEM, matched for follow-up duration, was enrolled to delineate disease-specific features.

**Results:**

Seven patients were diagnosed with Allgrove syndrome at our institution over the past 16 years, and all of them had homozygous or compound heterozygous mutations in the *AAAS* gene. The patients presented with alacrima from infancy developed adrenal insufficiency during childhood and were diagnosed with Allgrove syndrome at a mean age of 17. Compared to 12 matched idiopathic achalasia patients, Allgrove patients exhibited a significantly earlier onset of achalasia symptoms (mean age: 12.5 vs. 33.8 years, *p* = 0.004), with similar intervals from symptom onset to intervention. Interestingly, despite comparable objective severity based on manometric and endoscopic assessments, Allgrove patients reported significantly lower subjective symptom scores (Eckardt score 5.3 vs. 7.6, *p* = 0.046). Over a median follow-up of 5.8 years, the mean Eckardt score decreased from 5.3 to 0.8 in Allgrove patients and from 7.6 to 1.9 in idiopathic achalasia patients at the last follow-up.

**Conclusion:**

Allgrove syndrome should be taken into consideration in patients with early-onset achalasia. POEM provides effective and sustained symptom relief for achalasia in Allgrove syndrome, with a favorable safety profile.

## 1 Introduction

Allgrove syndrome (also known as triple A syndrome) is a rare autosomal recessive disorder, classically defined by the triad of adrenal insufficiency, alacrima, and achalasia. Since its initial description in 1978 by Allgrove *et al.* in two pairs of siblings, over 200 cases have been documented worldwide, and the knowledge regarding Allgrove syndrome has been expanding (1). It is recognized to be caused by homozygous or compound heterozygous mutations in the *AAAS* gene on chromosome 12q13 (2, 3). The *AAAS* gene consists of 16 exons and encodes the ALADIN protein (ALacrima, Achalasia, aDrenal Insufficiency, and Neurologic disorder), an assembly regulator and a vital component of nuclear pore complexes. Mutant ALADIN proteins are mislocalized to the cytoplasm, interfere with the normal assembly of nucleoporins, and consequently disrupt nucleocytoplasmic trafficking (4). However, the exact mechanisms underlying the specific organ involvement and the marked clinical variability observed in Allgrove syndrome remain unclear. Consequently, treatments are exclusively symptom-directed: Corticosteroid replacement for adrenal insufficiency, artificial tears for alacrima, and myotomy or dilation for achalasia. Unlike the well-established efficacy and accessibility of corticosteroid and artificial tear therapy, the benefits and risks of myotomy, particularly peroral endoscopic myotomy (POEM), for achalasia in Allgrove syndrome remain to be elucidated.

Achalasia is a rare esophageal motility disorder, with an estimated global pooled incidence of 0.78 cases per 100,000 person-years (5). The underlying etiology remains unclear but is thought to involve the loss of inhibitory myenteric neurons that regulate the distal esophagus and lower esophageal sphincter (LES) relaxation (6). Genetic factors, dysregulated autoimmunity, and viral infection might contribute to the pathogenesis of achalasia (7, 8). Clinical symptoms including dysphagia, regurgitation, vomiting, and non-cardiac chest pain are noted in achalasia patients (9). The diagnosis of achalasia is primarily based on high-resolution esophageal manometry (HREM), which also allows the classification of the disorder into three subtypes according to the Chicago Classification version 4.0 (10). All subtypes of achalasia exhibit impaired esophagogastric junction relaxation, while types I to III are characterized by absent pressurization, pan pressurization, and spastic contractions, respectively (11).

Despite different etiologies, treatments for achalasia aim to attenuate LES tension and facilitate the effective emptying of the esophageal lumen, even in the absence of peristalsis. The effects of calcium channel blockers and nitrates in achalasia are unreliable and temporary (6). Endoscopic botulinum toxin injection and pneumatic dilation provide symptom relief for only a few months up to a couple of years. Therefore, myotomy is recognized as the most definitive and permanent therapy for idiopathic achalasia (IAC). In recent years, peroral endoscopic myotomy (POEM) has emerged as a first-line treatment for idiopathic achalasia, offering durable symptom relief with high efficacy and safety, even in pediatric cases (6, 12–14). However, achalasia associated with Allgrove syndrome may follow a distinct clinical course, potentially influencing both disease behavior and treatment outcomes. Whether POEM offers comparable and durable benefits in these patients has not been systematically evaluated.

In this single-center cohort study, we summarized the clinical and genetic spectrum of seven patients with Allgrove syndrome (Allgrove patients) treated at our institution. The long-term efficacy of POEM in Allgrove patients was also explored in comparison with a matched cohort of patients with idiopathic achalasia (IAC).

## 2 Materials and methods

### 2.1 Study design and patients

This retrospective study included all patients diagnosed with Allgrove syndrome between 2009 and 2025 at Peking Union Medical College Hospital, the national rare disease center in China. The diagnosis of Allgrove syndrome was suspected when the patient presented with at least two symptoms of the classic triad (alacrima, adrenal insufficiency, and achalasia) and was further supported by the identification of *AAAS* gene mutations. Potential Allgrove patients were identified from the medical records system if they had a confirmed or suspected diagnosis of Allgrove syndrome, or at least two confirmed diagnoses among alacrima, adrenal insufficiency, and achalasia. Written informed consent was obtained from each patient or their guardian, and the study was approved by the ethics committee of our institution.

Clinical data, including demographic information, family history, symptom severity, and hormone measurements, were retrieved from the hospital information system or acquired through follow-up telephone interviews. Allgrove patients in our cohort underwent POEM between 2017 and 2021. To further characterize the features of achalasia in Allgrove patients and their responses to POEM, IAC patients who underwent POEM during the same period were chosen as a comparator cohort. IAC patients with follow-up information were selected from our endoscopy center records and matched to Allgrove patients by follow-up duration at a 1:3 ratio. A total of 12 IAC patients were randomly selected from 96 IAC patients who underwent POEM from 2017 to 2021 at our institution. The sex distribution and age were comparable between the sampled patients and the general population, confirming the randomness of sampling and the reliability of the results. Clinical characteristics, such as disease onset, duration, severity, and symptom relief following POEM, were compared between the Allgrove patients and the 12 IAC patients.

### 2.2 Gene analysis

For DNA analysis, blood samples were collected from the patients. Sanger sequencing of the *AAAS* gene or whole-exome sequencing was performed to identify mutations, as previously described (15).

### 2.3 Assessment of achalasia severity

Symptoms were quantified using the Eckardt scoring system, which evaluates the four major symptoms of achalasia—dysphagia, retrosternal pain, regurgitation, and weight loss—on a scale of 0–3 (16). The cumulative Eckardt score ranges from 0 to 12, with higher scores indicating more severe symptoms and scores ≤3 indicating clinical remission.

Endoscopic severity was assessed in four major aspects: Esophageal contents, lumen dilation, resistance at the lower esophageal sphincter (LES), and stasis (17). Contents (no contents, lipids retention, and food or pill retention in the esophagus), lumen dilation (normal caliber, dilated lumen, and severely dilated lumen without axial orientation), and resistance (absence of resistance, mild resistance, and moderate or severe resistance of the LES during scope passage) were graded on three levels, while stasis was evaluated as either absent or present.

Barium esophagram was also performed, with maximum dilation diameter and tortuosity evaluated by experienced radiologists. HREM was performed using an intra-esophageal pressure catheter with four pressure sensors positioned 5, 10, 15, and 20 cm above the upper margin of the LES. Baseline LES pressure was recorded for 5 min, and integrated relaxation pressure (IRP) was measured during water swallows. All manometric data were analyzed and classified based on the Chicago Classification version 4.0 (10).

### 2.4 POEM and follow-up

All POEM procedures were performed by experienced gastroenterologists using a standard technique (18). Endoscopy, HREM re-examination, and Eckardt scores 1 year after POEM were retrieved from our medical records system, and the most recent Eckardt scores were obtained via telephone interviews.

### 2.5 Statistics

Statistical analysis was conducted using GraphPad Prism version 9.0. Data were presented as mean ± standard deviation (SD) or median (25th and 75th percentiles) for continuous variables depending on the normality of their distribution. Categorical variables were summarized as frequencies and percentages. All data were tested for normality using the Shapiro–Wilk test. If the data were normally distributed, Student’s *t-*test was used to compare the two groups. Otherwise, non-parametric tests (such as the Mann–Whitney U test) were used. Differences in categorical variables were assessed using Fisher’s exact test. A *p*-value of <0.05 was considered statistically significant.

## 3 Results

### 3.1 Patient characteristics and genetic findings

Over a 16-year period (from 2009 to 2025), seven patients with genetically confirmed Allgrove syndrome were identified at Peking Union Medical College Hospital. All Allgrove patients were male, and six of them presented with the complete triad of symptoms (Table 1). Patient 7 also presented with orthostatic hypotension, indicating autonomic dysfunction. However, no neurological manifestations were noted in other patients. Alacrima was present in all Allgrove patients from infancy. Manifestations of adrenal insufficiency, including skin pigmentation and general weakness, presented at an average age of 6 years, with noted low serum cortisol and elevated adrenocorticotropic hormone levels. Achalasia symptoms in Allgrove patients resembled those observed in idiopathic achalasia. Moreover, one patient (patient 3) remained asymptomatic for achalasia at age 15 at the last follow-up. The mean age at definitive diagnosis of Allgrove syndrome in our cohort was 17 years, with the longest delay being 20 years from the onset of the complete symptom triad.

**Table 1:** Demographic and clinical characteristics of Allgrove patients.

Patients	Sex	Onset age (yrs.)			Age at diagnosis (yrs.)	Gene mutations	Consanguineous parents	Adrenal function measurements	
		Alacrima	Adrenal insufficiency	Achalasia				ACTH (pg/ml, <46)	Cortisol (μg/dL, 4–22.3)
1	M	Newborn	4	2	6	*AAAS* c.577C > T p.Q193* Het, c.1062_1063insAC p.S355T fs*62 Het	−	>1,250	1.8
2	M	Newborn	5	14	19	*AAAS* c.1062_1063insAC p.S355Tfs*62 Homo	−	1,133	1.6
3	M	Newborn	5	−	5	*AAAS* c.1062_1063insAC p.S355Tfs*62 Homo	−	1,149	<0.5
4	M	Newborn	5	22	27	*AAAS* c.771delG p.A258Gfs*33 Homo	N/A	241	3.6
5	M	Newborn	11	3	17	*AAAS* c.638G > A p.C213Y Het, c.1373delT p.I458T fs*93 Het	−	1,139	2.2
6	M	Newborn	11	11	31	*AAAS* c.908 T > C p.L303P Het, c. 580C > T p.R194X Het	−	605	0.8
7	M	Newborn	2	11	11	*AAAS* c.1332-2A > G Homo	+	>1,250	1.0

M, male; N/A, not available; −, symptoms not yet appeared or negative for consanguineous parents; +, positive; ACTH, adrenocorticotropic hormone.

**Figure 1:**
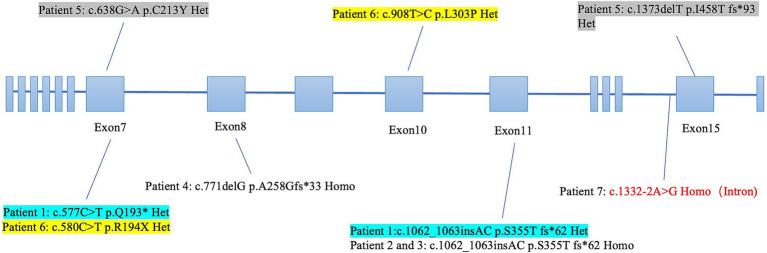
*AAAS* mutation site distribution in Allgrove patients. The *AAAS* gene consists of 16 exons, and seven Allgrove patients carried overlapping mutations. The most frequent mutation was *AAAS* c.1062_1063insAC, identified in three patients. The most common mutation type was frameshift. Patient 7, coming from a consanguineous family, carried the only mutation located in an intronic region.

All seven Allgrove patients carried either homozygous (*n* = 4) or compound heterozygous (*n* = 3) mutations in the *AAAS* gene (Figure 1). In total, nine mutations were identified in our cohort, localized at exons 7–15. The most frequent mutation was *AAAS* c.1062_1063insAC, which was present in three patients. The most common mutation type was frameshift, which caused protein truncation, and there was also one patient carrying a mutation in an intronic region. In accordance with the genetic nature of Allgrove syndrome, several patients had a family history of the disease. Patient 7 came from a consanguineous family, patients 2 and 3 were brothers, and patient 6 had a brother with similar symptoms and the same *AAAS* gene mutation, who declined formal evaluation.

### 3.2 Comparative analysis of achalasia features and POEM outcomes

Among the six Allgrove patients who presented with achalasia symptoms, two patients (patients 1 and 7) underwent surgical myotomy and four patients underwent POEM. To investigate the characteristics of achalasia and the efficacy of POEM in Allgrove syndrome, these four Allgrove patients were matched with 12 patients with idiopathic achalasia who underwent POEM at our institution during the same period (Table 2). The POEM-treated Allgrove cohort at our institution was exclusively male, while IAC patients had a more balanced sex distribution.

**Table 2:** Comparative analysis of achalasia features and POEM outcomes between Allgrove patients and idiopathic achalasia patients.

Characteristics	Allgrove patients (*n* = 4)					IAC patients (*n* = 12)	*P*
	2	4	5	6	Summary		
Sex (Male, %)	Male	Male	Male	Male	100%	50%	
Onset age of Achalasia (Mean ± SD, yrs.)	14	22	3	11	12.5 ± 7.9	33.8 ± 11.4	0.004
Duration of Achalasia (Mean ± SD, yrs.)	5	5	14	20	11.0 ± 7.3	7.7 ± 5.2	0.336
Achalasia subtype (I/II/III)	II	II	I	I	2/2/0	8/4/0	0.604
Before POEM							
Eckardt score (Mean ± SD)	4	5	7	5	5.3 ± 1.3	7.6 ± 2.0	0.046
Manometry							
IRP4s (Mean ± SD, mmHg)	30.6	20.6	34	12.9	24.5 ± 9.6	21.2 ± 7.7	0.516
Endoscopic evaluation							
Contents (absent or liquid/solid retention)	Absent	Liquid	Liquid	Solid	1/3	2/10	>0.999
Dilation (absent, mild/moderate, or severe)	Mild	Mild	Severe	Severe	2/2	5/7	>0.999
Resistance (absent, mild/moderate, or severe)	Mild	Mild	Moderate	Moderate	2/2	5/7	>0.999
Stasis (absent or present)	Absent	Absent	Absent	Present	3/1	5/7	0.569
Barium esophagram							
Maximum diameter (Mean ± SD, cm)	2.4	3.6	2.7	5.1	3.5 ± 1.2	4.1 ± 1.6	0.496
Tortuosity (absent, mild/moderate, or severe)	Moderate	Moderate	Mild	Mild	2/2	5/5	>0.999
1 year after POEM							
Eckardt Score (Median, 25, 75%)	1	1	2	2	1.5 (1.0, 2.0)	1.0 (1.0, 2.0)	0.884
4–8 years after POEM							
Eckardt score (Mean ± SD)	0	0	1	2	0.8 ± 1.0	1.9 ± 1.4	0.158

IAC, idiopathic achalasia; POEM, peroral endoscopic myotomy; IRP4s, integrated relaxation pressure; N/A, not available.

Symptoms of achalasia appeared significantly earlier in Allgrove patients than in IAC patients (mean age 12.5 ± 7.9 vs. 33.8 ± 11.4 years, *p* = 0.004), while the duration before POEM was comparable between the two groups (11.0 ± 7.3 vs. 7.7 ± 5.2 years, *p* = 0.336). Moreover, the two groups also exhibited a similar distribution of achalasia subtypes.

Regarding baseline achalasia severity, Allgrove patients presented with a significantly lower pre-POEM Eckardt score compared to IAC patients (5.3 ± 1.3 vs. 7.6 ± 2.0, *p* = 0.046), indicating milder subjective discomfort. Nevertheless, objective measurements of achalasia severity were comparable between the two groups. First, no significant differences were observed in integrated relaxation pressure (IRP4s) measured by HREM (24.5 ± 9.6 vs. 21.2 ± 7.7 mmHg, *p* = 0.516). Second, endoscopic evaluation of Allgrove patients and IAC patients across four dimensions (contents, dilation, resistance, and stasis) showed similar severity ratings, with esophageal dilation and LES resistance observed in all patients. Finally, dilation severity, as indicated by maximum diameter and tortuosity on barium esophagram, was also comparable between the two groups (3.5 ± 1.2 vs. 4.1 ± 1.6 cm, *p* = 0.496).

### 3.3 Post-POEM follow-up and efficacy

Postoperative follow-up confirmed the efficacy of POEM in Allgrove syndrome. In total, two Allgrove patients underwent repeat HREM 1 year after POEM, both showing a marked decrease in IRP4s (30.6 vs. 10.4, 20.6 vs. 2.8 mmHg). In addition, three Allgrove patients underwent follow-up endoscopy, demonstrating alleviation in esophageal content retention, dilation, and LES resistance. A total of four IAC patients underwent repeat HREM 1 year after POEM, all showing a significant decrease in IRP4s (39.3 vs. 1.1, 19.9 vs. 2.8, 25.1 vs. 1.1, 17.6 vs. 7 mmHg). Follow-up endoscopy in four IAC patients also confirmed amelioration of achalasia (data not shown). Furthermore, both Allgrove and IAC patients maintained symptomatic relief, with significantly decreased but comparable Eckardt scores at 1-year and 4–8-year follow-up, confirming the efficacy of POEM in the two groups.

## 4 Discussion

This long-term comparative cohort study provides several clinically relevant insights into the diagnosis and management of achalasia in Allgrove syndrome. Our key findings are as follows: (1) a substantial diagnostic delay existed in Allgrove syndrome, underscoring the need for heightened clinical awareness; (2) although achalasia in this syndrome presented with early onset, we observed a notable dissociation between subjective symptom reporting and objective disease severity; and (3) POEM provided safe, effective, and durable symptom relief for achalasia in Allgrove syndrome, with outcomes comparable to idiopathic achalasia over a median follow-up of 5.8 years.

Herein, we assembled a valuable group of Allgrove patients with confirmed *AAAS* mutations. Alacrima was present in all patients since infancy, and adrenal insufficiency symptoms appeared earlier than those of achalasia. Identifying *AAAS* mutations was crucial for the final diagnosis of Allgrove syndrome, and some mutations identified in our cohort were reported for the first time (*AAAS* c.638G > A, c.1373delT, and c. 908 T > C). However, the relationship between specific *AAAS* mutation sites and phenotypic variability remains to be elucidated (19–22). Overall, the mean age at diagnosis of Allgrove syndrome in our cohort was 17 years, with a mean delay of 5.5 years from the onset of the triad symptoms. Greater awareness of this rare syndrome is essential for achieving timely diagnosis. Alacrima, as an early warning sign of Allgrove syndrome, should be evaluated with greater attention. Increasing awareness of Allgrove syndrome among endocrinologists and gastroenterologists would facilitate its recognition and enable timely diagnosis.

Achalasia is one of the major symptoms of Allgrove syndrome, and idiopathic achalasia itself is a rare esophageal motility disorder. Pediatric achalasia has an even lower incidence, estimated at0.1–0.18 cases per 100,000 person-years (23). Achalasia in Allgrove patients is often classified as pediatric or familial achalasia and typically presents at an earlier age than idiopathic achalasia, which usually occurs between 20 and 40 years of age (24). In an Algerian cohort of 1,256 achalasia patients, 18 families comprising 41 achalasia patients were identified; among them, 34 patients from 15 families were diagnosed with Allgrove syndrome (25). In an Asian cohort of 1,115 patients, familial achalasia and Allgrove syndrome were observed at lower frequencies of 0.63 and 0.18%, respectively (24). Pediatric achalasia symptoms include vomiting, progressive dysphagia, recurrent pneumonia, nocturnal cough, feeding problems, and failure to thrive (26). These symptoms are non-specific; therefore, gastroenterologists should remain vigilant when managing pediatric achalasia and consider the possibility of Allgrove syndrome.

Treatment options for achalasia have advanced considerably in recent years, ranging from pharmacologic therapies such as nitrates to endoscopic interventions including botulinum toxin injection and pneumatic dilation, as well as surgical myotomy and POEM. Therapeutic efficacy has become more reliable and sustained, while invasiveness has been significantly reduced. Histologically, a decrease in ganglion cells and lymphocytic infiltration are common findings in achalasia patients, along with mild fibrosis (27). Specifically, a histopathological study of 10 Allgrove patients showed enhanced fibrosis of the intermuscular plane, tremendous reductions in myenteric ganglia and neurons, and a lack of neuronal nitric oxide synthase expression in the cardia (28). Despite inconsistent histological changes, myotomy is still the preferred intervention for both groups (29). Among the six Allgrove patients in our cohort who presented with achalasia symptoms, two underwent surgical myotomy in 2010 and 2015, while the remaining four underwent POEM at our hospital from 2017 to 2021.

To clarify the clinical features of achalasia in Allgrove patients and their response to POEM, 12 IAC patients were enrolled, matched for follow-up duration. As expected, we confirmed significantly earlier onset of achalasia and earlier ages at POEM in Allgrove patients. Achalasia in Allgrove patients appeared during childhood or early adolescence, while IAC patients experienced the achalasia symptoms in middle age, as reported previously (24). Allgrove patients reported less clinical discomfort, as indicated by significantly lower Eckardt scores. However, achalasia subtype distribution, IRP4s levels, endoscopic assessments, and radiography measurements were all comparable between Allgrove and IAC patients, objectively confirming similar disease severity in both groups at the time of POEM. We hypothesize that the dissociation between subjective symptom reporting and objective disease severity in Allgrove patients might be attributable to earlier disease onset, enhanced adaptability, or differences in symptom expression.

The short-term efficacy of POEM in Allgrove patients has been reported in a few case reports. The youngest reported Allgrove patient to undergo POEM was only 7 years old, demonstrating both the efficacy and safety of POEM (30). In addition, three adolescent Allgrove patients further confirmed the effectiveness of POEM over a 2–4-month follow-up period (31). Moreover, in a 31-year-old male with a 4-year history of achalasia symptoms, achalasia amelioration after POEM was still evident at 1-year follow-up (32). Our study followed the four Allgrove patients who underwent POEM for 4–8 years and, for the first time, verified persistent long-term alleviation of their achalasia symptoms. During this follow-up period, we observed significant and sustained decreases in IRP4s, endoscopic findings, and Eckardt scores in these patients after POEM. Long-term symptom relief in IAC patients after POEM has also been documented previously (6). Moreover, the Eckardt score in IAC patients exhibited a trend toward relapse over time, while Allgrove patients maintained consistently low scores during follow-up, suggesting an even more durable effect of POEM in Allgrove patients.

However, this single-center retrospective study has certain limitations. Due to the retrospective design, some patients did not complete post-operative HRM examinations, limiting the objective assessment of POEM efficacy. Therefore, further studies with standardized follow-up protocols are needed to elucidate the clinical characteristics and therapeutic responses of Allgrove syndrome. Awareness of this rare disease should be increased among physicians across multiple medical centers to ensure its proper diagnosis and management.

## 5 Conclusion

In conclusion, this study summarizes the clinical and genetic characteristics of a cohort of Allgrove patients diagnosed at our institution over the past 16 years. Mutations in the *AAAS* gene were noted in all patients who developed achalasia earlier and underwent POEM at a younger age compared to idiopathic achalasia patients. Through follow-up, both the short-term and long-term efficacy of POEM were demonstrated in Allgrove patients, providing evidence to support optimized management of Allgrove syndrome.

## Data Availability

The data analyzed in this study is subject to the following licenses/restrictions: the datasets used and/or analyzed during the current study are available from the corresponding author on reasonable request. Data cannot be openly shared due to participant privacy. Requests to access these datasets should be directed to XL, lixiaoqing20060417@126.com.
